# 30-day readmission rate in pediatric otorhinolaryngology inpatients: a retrospective population-based cohort study

**DOI:** 10.1186/s40463-021-00536-8

**Published:** 2021-09-20

**Authors:** Katharina Geißler, Wido Rippe, Daniel Boeger, Jens Buentzel, Kerstin Hoffmann, Holger Kaftan, Andreas Mueller, Gerald Radtke, Orlando Guntinas-Lichius

**Affiliations:** 1 Department of Otorhinolaryngology, Jena University Hospital, Am Klinikum 1, 07747 Jena, Germany; 2 Department of Otorhinolaryngology, SRH Zentralklinikum, Suhl, Germany; 3 Department of Otorhinolaryngology, Südharz-Krankenhaus gGmbH, Nordhausen, Germany; 4 Department of Otorhinolaryngology, Sophien- Und Hufeland-Klinikum, Weimar, Germany; 5 Department of Otorhinolaryngology, HELIOS-Klinikum, Erfurt, Germany; 6 Department of Otorhinolaryngology, SRH Wald-Klinikum, Gera, Germany; 7 Department of Otorhinolaryngology, Ilm-Kreis-Kliniken, Arnstadt, Germany

**Keywords:** Readmission rate, Pediatric, Otorhinolaryngology, Rehospitalization, Healthcare research

## Abstract

**Objectives:**

Analysis of frequency and reasons for planned and unplanned 30-day readmission in hospitalized pediatric otorhinolaryngology patients using German Diagnosis Related Group (G-DRG) system data.

**Methods:**

A retrospective population-based cohort study in Thuringia, Germany, was performed for the year 2015 with 2440 cases under 18 years (55.6% male) out of a total number of 15.271 inpatient cases. The majority of pediatric patients were from 2 to 5 years old (54.5%). The most frequent diagnoses were hyperplasia of adenoids or/and tonsils (26.6%). 36 cases (1.5%) experienced readmission within 30-days.

**Results:**

30-day readmission was planned in 9 cases (25% of all readmission) and was unplanned in 27 cases (75%). The median interval between index and readmission treatment was 8 days. Postoperative bleeding after adenoidectomy, tonsillotomy/tonsillectomy or tracheostomy (33.4%) and infectious complications after surgery like acute otitis media, abscess formation or fever (36.2%) were the most frequent reasons for 30-day readmission. Compared to adults treated in 2015 in Thuringia, the readmission rate was higher in adult patients (8.9%) than in this pediatric cohort. In contrast to children, readmissions in adults were mainly planned (65.1%) with a different spectrum of underlying diseases and reasons for readmission.

**Conclusion:**

The 30-day readmission rate seemed to be lower for pediatric otolaryngology patients compared to adult patients. Unplanned readmissions dominated in pediatric patients, whereas planned readmissions dominated in adults.
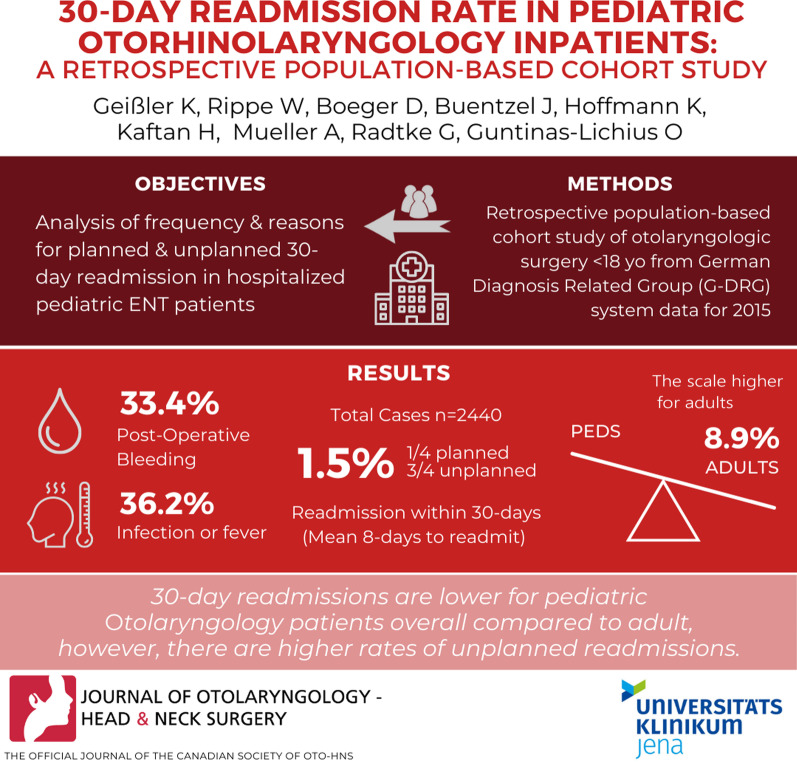

**Supplementary Information:**

The online version contains supplementary material available at 10.1186/s40463-021-00536-8.

## Introduction

Hospital readmissions are an outcome measure used in heath service research as a metric for health care quality [1]. Potentially avoidable readmissions can be the consequence of an adverse event or a too early discharge of a prior hospitalization [2]. One of the most widely used tools for reimbursing inpatient health services around the world is Diagnosis-Related Groups (DRGs) [3]. In German DRG (G-DRG) system, readmissions for the same cause within 30 days after discharge are reimbursed by the original DRG and receive no additional funds [4].

Some studies have published 30 day readmission rates of specific pediatric surgeries such as pediatric bone and soft tissue sarcoma surgery [5], urachal remnants [6], congenital heart surgery [7], esophageal atresia [8] and burn injured patients [9], but not in an overall pediatric otorhinolaryngological cohort.

Recently, we have published a population-based study for analyzing the 30-day unplanned readmission rate in otorhinolaryngology patients [10]. The 30-day readmission was planned in 4.9% and unplanned in 2.8%. The most frequent reasons for readmission were: Need for non-surgical therapy (31.2%), need for further surgery (26.3%), post-surgical complaints (16.9%), and recurrence of primary complaints (10.7%). A multivariate analysis revealed that discharge due to patient's request against medical advice was a strong independent factor with high risk for unplanned readmission. Surgery at index admission was the second important independent risk factor for unplanned readmission.

As a next step, we investigated in the present study the differences in frequency and reasons for readmission between children under 18 years and adults in 2015 in Thuringia. Thuringia is a territorial state in Germany with approximately 2.2 million habitants. There are eight hospitals with departments of otorhinolaryngology, which have built an ideal network to improve health services research in the field of otorhinolaryngology [11, 12]. We hypothesized that children admitted under the otolaryngology service often suffer from less serious diseases such as adenoid hyperplasia or chronic otitis media in comparison to adults. Therefore, we assumed that they would have to be re-admitted less frequently.

## Material and methods

A retrospective analysis was performed in seven of eight Thuringian hospitals that have a department of otorhinolaryngology. The eighth hospital did not take part. These seven hospitals covered about 90% of all inpatient otorhinolaryngology cases in Thuringia.

All otorhinolaryngology patients with inpatient treatment in 2015 were included. Outpatients and day-care patients were excluded. The patients were screened through the hospital information systems of the seven participating hospitals. The patient ID was used to identify all patients with readmission within 30 days. These patients built the group of primary interest (readmission group). The other patients built the group of patients without 30-day readmission (no readmission group). To characterize the readmission group in more detail, and especially to identify the patients with planned versus unplanned readmission, the patients’ charts of all patients with 30-day readmission were revisited. A planned readmission was defined as a readmission that was planned during the index admission. Examples for a planned readmission were a post-operative interval endoscopy in case of choanal atresia, the removal of a tamponade out of the ear canal after ear surgery, the removal of branchial cyst or thyroglossal duct cyst after initial infection or in case of recurrence. Even a surgery like cochlea implantation after an initial admission for diagnostic workup with brainstem evoked response audiometry (BERA) or electrocochleography was evaluated as planned readmission. All other patients without planned readmission were defined as unplanned readmissions. Follow-up examinations longer than 30 days were not evaluated. Variables collected from readmission and no readmission group were gender, age, diagnosis at first inpatient treatment and every readmission, duration of stay, reason for readmission, time between end of inpatient treatment and readmission.

The primary outcome was to analyze frequency and reasons for 30-day readmission in children. Secondary outcome measurements were the diagnosis at first inpatient treatment, the duration of stay and the time until readmission. Finally, these results were compared to adults treated in 2015 at departments of otorhinolaryngology in Thuringia as published recently [10].

### Statistical analyses

Patient demographics and outcome variables, statistical t-tests for unpaired samples, chi-square-test and univariate variance analysis with Bonferroni correction were performed with IBM SPSS statistics software (IBM SPSS Statistics for Windows, Version 21, Chicago, IL). Data are presented as frequencies or mean ± standard deviation (SD) if not otherwise indicated. The level of significance was set to p < 0.05.

## Results

30-day readmission was planned in 9 cases (25%) and unplanned in 27 cases (75%). The median interval between index inpatient treatment and readmission was 8 days. Postoperative bleeding after adenoidectomy, tonsillotomy/tonsillectomy or tracheostomy (33.4%) and infectious complications after surgery like acute otitis media, abscess formation or fever (36.2%) were the most frequent reasons for 30-day readmission.

### Comparison of children without and with 30-day readmission

In 2015, 36 cases of underage patients (1.5%) experienced readmission within 30-days. The univariate comparisons between patients without and with 30-day readmission are summarized in Table 1. Children with 30-day readmission had a significant longer index inpatient stay (4.3 ± 2.2 days) than children without 30-day readmission (2.5 ± 2.0 days, p < 0.0001). Otherwise, no significant differences were obvious.

**Table 1:** Comparison of the group of children with 30-day readmission to the group of children without 30-day readmission

Parameter	All	Readmission		No readmission		p
	N	N	%	N	%	
All	2440	36	1.5	2404	98.5	
*Gender*						0.73
Male		19	52.8	1337	55.6	
Female		17	47.2	1067	44.4	
*Index treatment DRG-partition*	2427					0.15
Surgical		28	80.0	2104	88.0	
Medical		7	20.0	288	12.0	
Missing	13	1		12		

	Mean ± SD	Mean ± SD		Mean ± SD		
Age, years	5.8 ± 4.4	6.0 ± 5.1		5.8 ± 4.4		0.77
Primary treatment duration, days	2.5 ± 2.0	4.3 ± 2.2		2.5 ± 2.0		< 0.0001

*DRG* diagnosis related group, *SD* standard deviation

### Comparison of children with planned and unplanned 30-day readmission

The unplanned 30-day readmission rate was higher in female than male children (p = 0.01, Table 2). Surgical treatment during the index treatment was more frequently found in children with unplanned compared to children with planned 30-day readmission (p = 0.03).

**Table 2:** Comparison of the group of children with planned and unplanned 30-day readmission

Parameter	All	Planned readmission		Unplanned readmission		p
	N	N	%	N	%	
All	36	9	25.0	27	75.0	
*Gender*						0.01
Male		8	88.9	11	40.7	
Female		1	11.1	16	59.3	
*Index treatment DRG-partition*						0.03
Surgical		5	55.6	23	88.5	
Medical		4	44.4	3	11.5	
Missing	1	0		1		

	Mean ± SD	Mean ± SD		Mean ± SD		
Age, years	6.0 ± 5.1	3.7 ± 3.1		6.7 ± 5.5		0.12
Primary Treatment treatment duration, days	4.3 ± 2.2	3.4 ± 2.1		4.5 ± 2.1		0.28

*DRG* diagnosis related group, *SD* standard deviation

## Discussion

To our knowledge, this is the first investigation of the readmission rate in an unselected population-based series of pediatric inpatients with otorhinolaryngological diseases. Recently, we published data on the readmission rates of adults [10]. Compared to adults, the 30-day-readmission rate of underage patients was significantly lower in children (1.5%) than in adults (8.9%). Readmission was more often planned in adults compared to children. A detailed comparison between children and adults is shown in Additional file 1: Table S1.

Children with 30-day readmission had a significantly longer first inpatient stay than children without 30-day readmission. Children with planned 30-day readmission were more frequently male. Reasons for unplanned 30-day readmission were frequently surgical procedures.

The limitation of this study was the retrospective design not allowing a causal analysis of risk factors for readmission, and especially for unplanned readmissions. The annual number of readmissions in children, especially of unplanned admissions, was low. Therefore, the possibilities of meaningful comparisons of pediatric patients with planned versus unplanned readmissions were limited. A longer observation period of 5 years would have been useful to identify independent risk factors for unplanned readmissions. As all patients within the study period were included, a selection bias could be ruled out. All other important sources of bias (observation bias, confirmation bias) could be neglected in the chosen methodological setting. Missing data could be a source of bias. Regarding this, we used the method of listwise deletion based on the assumption of missing at random. An important strength of the present study was the examination of all under age inpatients, and of the adults in a recent publication [10], of one year in one federal state to get a representative cross-section of the population.

Zheng et al. investigated the unplanned readmission rates and identified risk factors of unplanned readmissions in pediatric general surgical specialties [13]. Of the 3263 patients who underwent surgery and discharge, 176 (9%) were unplanned readmissions. The most common surgery related to readmission was appendectomy, and the most common causes for readmission were associated with treatment of gastrointestinal complaints or complications. Emergency surgery, major complications and the initial hospital length of stay were independent risk factors for readmission. In our study, a longer first inpatient treatment was observed more frequently in children with readmission. Independent risk factors could not be calculated because of the low number of readmissions in children. Major complications like bleeding or infection after surgery were frequent reasons for readmission, especially of unplanned readmission. Children with long inpatient treatment should be examined again short-termly after dismissal to avoid inpatient readmission.

Gilani and Bhattacharyya investigated revisit rates for pediatric tonsillectomy in departments of ambulatory surgery, emergency departments and inpatient hospital settings [14]. The study included 33,611 children. Revisit rates were significantly higher when the patient was discharged late during the day. Also, late afternoon surgery was significantly associated with higher revisit rates in case of bleeding, fever, nausea, vomiting, dehydration or pain. In our study, the exact daytime of surgery and discharge was not considered, but may potentially be important for further studies.

Lindquist et al. analyzed age-related causes of visits to emergency department after pediatric adenotonsillectomy [15]. 5225 patients were identified, with an overall late complication rate of 12.8%. There was no difference in the 30-day emergency department readmission rate for children under the age of three, although children under the age of two were more likely to present to the emergency department. There was a significantly higher risk of dehydration for children under the age of 4 years, and a significantly higher bleeding risk and need for reoperation for children over the age of six. In our investigation, the 30-day emergency department readmission rate was not analyzed. It could be important to analyze the 30-day emergency department readmission, because sometimes a recurrent emergency department admission could be contributed to an inpatient readmission.

Denning et al. analyzed the effect of outpatient follow-up after pediatric surgery [16]. Thirty-day emergency department visits and readmission rates were significantly lower in those patients with outpatient follow up than in those without (8.8% vs 12.7%, p = 0.04 and 3.7–11.0%, p < 0.001, respectively). Outpatient follow up was more beneficial in patients with inpatient procedures or longer hospitalization lengths than in the cohort of ambulatory patients. In our study, a standardized outpatient follow up was not performed and could therefore not be analyzed.

In comparison to adults, children in a department of otorhinolaryngology in Germany had significantly fewer readmissions. One possible reason is, that pediatric patients had less planned readmissions due to a lower incidence of severe diseases like head and neck cancer. 22.5% of adults had a readmission because of chemotherapy or complications of chemotherapy. No pediatric patient had a readmission because of this therapy. Furthermore, children had less often high risk surgery (children 1.5%; adults 11.4%) like tumor surgery and less often chronic diseases (children 5.3%; adults 21.2%). The main reason for readmission in pediatric patients were bleeding after surgery like adenoidectomy, tonsillotomy or tonsillectomy and abscess and postoperative wound healing disorder. To verify that these factors are predictors for planned or unplanned readmission, it would be worthwhile to repeat the study with a population from another federal state in Germany or another country with comparable health care system.

## Conclusion

The presented population-based analysis out of an 1 year series of unselected adult and pediatric otorhinolaryngology inpatient cases revealed that the 30-day readmission rate was lower for pediatric patients compared to adult patients. Unplanned readmissions dominate in pediatric patients, whereas planned readmissions dominate in adults. A planned outpatient examination could be useful in case of long inpatient treatment to avoid readmission.

## Supplementary Information

**Additional file 1: Table S1**. Comparison between 2440 cases of pediatric patients and 12.831 cases of adult patients from a recent publication [10]

## Data Availability

The original data and materials are available.

## References

[1] Jha AK, Orav EJ, Epstein AM (2009). Public reporting of discharge planning and rates of readmissions. New Eng J Med.

[2] Busato A, von Below G (2010). The implementation of DRG-based hospital reimbursement in Switzerland: A population-based perspective. Health Res Policy Syst.

[3] Hopfe M, Stucki G, Bickenbach JE, Prodinger B (2018). Accounting for what matters to patients in the G-DRG system: a stakeholder’s perspective on integrating funtioning information. Health Serv Insights.

[4] Geissler A, Scheller-Kreinsen D, Busse R, Busse R, Geissler A, Quentin W, Wiley M (2011). Germany: understanding G-DRGs. Diagnosis-related groups in Europe: moving towards transparency, efficiency and quality in hospitals.

[5] Gallaway KE, Ahn J, Callan AK (2020). Thirty-day outcomes following pediatric bone and soft tissue sarcoma surgery: a NSQIP pediatrics analysis. Sarcoma.

[6] Aylward P, Samson K, Raynor S, Cusick R (2020). Operative management of urachal remnants: an NSQIP based study of postoperative complications. J Pediatr Surg.

[7] Lushaj EB, Hermsen J, Leverson G, MacLellan-Tobert SG, Nelson K, Amond K, Anagnostopoulos PV (2020). Beyond 30 days: analysis of unplanned readmissions during the first year following congenital heart surgery. World J Pediatr Congenit Heart Surg.

[8] Quiroz HJ, Turpin A, Willobee BA, Ferrantella A, Parreco J, Lasko D, Perez EA, Sola JE, Thorson CM (2020). Nationwide analysis of mortality and hospital readmissions in esophageal atresia. J Pediatr Surg.

[9] Tapking C, Boson AL, Rontoyanni VG, Hundeshagen G, Kowalewski KF, Popp D, Houschyar KS, Zapata-Sirvent R, Branski LK (2019). A systematic review and meta-analysis of 30-day readmission rates following burns. Burns.

[10] Rippe W, Dittberner A, Boeger D, Buentzel J, Hoffmann K, Kaftan H, Mueller A, Radtke G, Guntinas-Lichius O (2019). 30-day unplanned readmission rate in otolaryngology patients: a population-based study in Thuringia, Germany. PLoS ONE.

[11] Mueller J, Boeger D, Buentzel J, Esser D, Hoffmann K, Jecker P, Mueller A, Radtke G, Geißler K, Bitter T, Guntinas-Lichius O (2015). Population-based analysis of tonsil surgery and postoperative hemorrhage. Eur Arch Otorhinolaryngol.

[12] Thomas K, Boeger D, Buentzel J, Esser D, Hoffmann K, Jecker P, Mueller A, Radtke G, Geißler K, Finkensieper M, Guntinas-Lichius O (2013). Pediatric adenoidectomy: a population-based regional study on epidemiology and outcome. Int J Pediatr Otorhinolaryngol.

[13] Zheng C, Zhou H, Zhu H, Chen B, Qiu L, Guo C (2019). Understanding unplanned readmissions for children undergoing surgery in a single pediatric general surgical department. BMC Pediatr BMC Pediatr.

[14] Gilani S, Bhattacharyya N (2020). Revisit rates for pediatric tonsillectomy: an analysis of admit and discharge times. Ann Otol Rhinol Laryngol.

[15] Lindquist NR, Feng Z, Patro A, Mukerji SS (2019). Age-related causes of emergency department visits after pediatric adenotonsillectomy at a tertiary pediatric referral center. Int J Pediatr Otorhinolaryngol.

[16] Denning NL, Glick RD, Rich BS (2020). Outpatient follow-up after pediatric surgery reduces emergency department visits and readmission rates. J Pediatr Surg.

