# Supplementary material for: 30-day readmission rate in pediatric otorhinolaryngology inpatients: a retrospective population-based cohort study
Source: J Otolaryngol Head Neck Surg. 2021 Sep 20;50:55. doi: 10.1186/s40463-021-00536-8 (PMC8454104; doi:10.1186/s40463-021-00536-8)
Supplement: Supplementary file 1 — Additional file 1: Table S1. Comparison between 2440 cases of pediatric patients and 12.831 cases of adult patients from a recent publication [10] [file 40463_2021_536_MOESM1_ESM.docx]

**Table S1: Comparison between 2440 cases of pediatric patients and 12.831 cases of adult patients from a recent publication [10].**

|  | **children** | | **adults** | | **p** |
| --- | --- | --- | --- | --- | --- |
| **Parameter** | **N** | **%** | **N** | **%** |  |
| **Gender** |  | |  | | <0.0001 |
| Female | 1084 | 44.4 | 5131 | 40.0 |  |
| Male | 1356 | 55.6 | 7700 | 60.0 |  |
| **30-day readmission** |  | |  | |  |
| Yes | 36 | 1.5 | 1137 | 8.9 | Yes vs. No <0.0001 |
| No | 2404 | 98.5 | 11692 | 91.1 |  |
| Planned | 9 | 25.0 | 743 | 65.1 | Planned vs. Unplanned <0.0001 |
| Unplanned | 27 | 75.0 | 399 | 34.9 |  |
| **Localization of the primary disease at the index admission** |  | |  | | <0.0001 |
| Pharynx/ oral cavity | 1733 | 71.0 | 2924 | 22.8 |  |
| Ear | 351 | 14.4 | 2702 | 21.1 |  |
| Paranasal sinus | 58 | 2.4 | 2046 | 15.9 |  |
| Larynx | 9 | 0.4 | 954 | 7.4 |  |
| Outside head and neck region | 49 | 2.0 | 817 | 6.4 |  |
| Face/ skin | 76 | 3.1 | 832 | 6.5 |  |
| Nose | 27 | 1.1 | 661 | 5.2 |  |
| Neck | 65 | 2.7 | 543 | 4.2 |  |
| Salivary gland | 14 | 0.6 | 530 | 4.1 |  |
| Trachea/ lung | 41 | 1.7 | 350 | 2.7 |  |
| Esophagus | 9 | 0.4 | 275 | 2.1 |  |
| Head/neck, not otherwise specified | 1 | 0.0 | 80 | 0.6 |  |
| Eye | 7 | 0.3 | 63 | 0.5 |  |
| Thyroid | - | - | 54 | 0.4 |  |
| **Reason for readmission** |  | |  | | <0.0001 |
| Bleeding after surgery/ tumor | 12 | 33.4 | 303 | 26.7 |  |
| Postoperative abscess/ wound healing disorder/ fever | 13 | 36,2 | 285 | 25.1 |  |
| Cochlea implantation | 3 | 8.3 | 25 | 2.2 |  |
| Dizziness after ear surgery | 3 | 8.3 | 10 | 0.9 |  |
| Chemotherapy and complications of chemotherapy | - | - | 255 | 22.5 |  |
| Epistaxis | - | - | 165 | 14.5 |  |
| Other | 5 | 14 | 90 | 7.9 |  |
| Missing | - | - | 2 | 0.0 |  |
|  | **Mean±SD** | | **Mean±SD** | |  |
| Age, years | 5.8±4.4 | | 55.0±17.9 | | <0.0001 |

ICD = International Classification of Diseases; SD = Standard deviation
